# PET imaging in healthy subjects and migraineurs suggests CGRP receptor antagonists do not have to act centrally to achieve clinical efficacy

**DOI:** 10.1186/1129-2377-1-S14-P224

**Published:** 2013-02-21

**Authors:** SGG Vermeersch, J de Hoon, B De Saint-Hubert, I Derdelinckx, K Serdons, G Bormans, T Reynders, R Declercq, I De Lepeleire, W Kennedy, R Blanchard, E Marcantonio, R Hargreaves, CC Li, S Sanabria, E Hostetler, A Joshi, J Evelhoch, K Van Laere

**Affiliations:** 1 Center for Clinical Pharmacology, Campus Gasthuisberg, University Hospitals Leuven (KU Leuven), Leuv, Belgium; 2 Nuclear Medicine Department, UZ and KU Leuven, Belgium; 3 Laboratory for Radiopharmacy, KU Leuven, Belgium; 4 Merck Sharp & Dohme (Europe) Inc., Brussels, Belgium; 5 Merck Research Laboratories, Upper Gwynedd PA, USA; 6 Merck Research Laboratories, West Point PA, USA

## 

Calcitonin gene-related peptide (CGRP) is a potent vasodilator and sensory neuropeptide implicated in the pathophysiology of migraine headache. CGRP receptor (CGRP-R) antagonists, including telcagepant, have shown clinical efficacy in treating migraine. CGRP-Rs are expressed in the CNS, particularly in the brainstem and cerebellum as well as in the periphery on vascular smooth muscle cells. To investigate whether central CGRP-Rs were likely to be involved in the anti-migraine effects of CGRP-R antagonists we examined central CGRP-R occupancy (CGRP RO) at an efficacious dose of telcagepant in healthy volunteers and in migraineurs during ictal and interictal periods using the novel PET tracer [11C]MK-4232. CGRP RO was evaluated in healthy subjects (n=3) at the lowest clinically efficacious dose (140 mg, PO) of telcagepant ~2h after dosing, coinciding with the time point of efficacy evaluation in clinical migraine studies. PET imaging showed only low CGRP RO (4% - 10%) which is within test-retest variability, suggesting that central activity is not a pre-requisite for anti-migraine efficacy of CGRP-R antagonists. A supratherapeutic telcagepant dose (1120 mg, PO) produced only moderate (43-58%, n=4) CGRP RO at ~Tmax (3h after administration). Subsequently, the possibility that brain penetration of telcagepant, a P-gp brain efflux pump substrate, may be increased in migraine patients or during migraine by blood brain barrier opening, was investigated by PET studies in the ictal and interictal periods in migraineurs (n=3) ~2 h after telcagepant dosing (140 mg, PO). Comparison of [11C]MK-4232 PET study results from ictal and interictal periods in migraineurs to healthy volunteers, suggests similar low RO and no significant differences between states. In conclusion, PET studies with the CGRP-R PET tracer [11C]MK-4232 after therapeutic doses of telcagepant in healthy volunteers and migraineurs suggest that central antagonism of CGRP-R is not necessary for therapeutic efficacy in migraine pain relief and that migraine pain is therefore at least in part peripheral in origin.
